# An optimized segmentation and quantification approach in microvascular imaging for OCTA-based neovascular regression monitoring

**DOI:** 10.1186/s12880-021-00546-y

**Published:** 2021-01-22

**Authors:** Sheng Wu, Shaowei Wu, Hui Feng, Zizhong Hu, Yejing Xie, Yun Su, Ting Feng, Li Li

**Affiliations:** 1 Medical Supplies Center, Chinese PLA General Hospital, Beijing, 100039 China; 2 School of Electronic and Optical Engineering, Nanjing University of Science and Technology, Nanjing, 210094 China; 3 Department of Ophthalmology, The First Affiliated Hospital of Nanjing Medical University, Nanjing, 210029 China; 4 Eye Hospital, Department of Ophthalmology, The Fourth School of Clinical Medicine, Nanjing, 210029 China; 5 Zhongda Hospital Affiliated to Southeast University, Nanjing, 210009 China

**Keywords:** OCTA, Blood vessel segmentation, Vessel extraction, Neovascularization, Diabetic retinopathy

## Abstract

**Background:**

Quantification of neovascularization changes in terms of neovascular complex (NVC) acquired from the optical coherence tomography angiography (OCTA) imaging is extremely important for diagnosis and treatment monitoring of proliferative diabetic retinopathy (PDR). However, only few vessel extraction methods have so far been reported to quantify neovascular changes in NVC with proliferative diabetic retinopathy PDR based on OCTA images.

**Methods:**

Here we propose an optimized approach to segment blood vessels, which is based on an improved vascular connectivity analysis (VCA) algorithm and combined with morphological characterization and elimination of noise and artifacts. The length and width of vessels are obtained in the quantitative assessment of microvascular network. The feasibility of the proposed method is further studied by a treatment monitoring and statistical analysis process, as we have monitored and statistically analyzed the changes of NVC based on sampled OCTA images of PDR patients (N = 14) after treatment by intravitreal injection of conbercept.

**Results:**

The proposed method has demonstrated better performance in accuracy compared with existing algorithms and can thus be used for PRD treatment monitoring. Following the PDR treatment monitoring study, our data has shown that from the 1st day to 7th day of treatment, the averaged (arithmetic mean) length of NVC has been substantially shortened by 36.8% (*P* < 0.01), indicating significant effects of treatment. Meanwhile, the averaged (arithmetic mean) width of NVC from the 1st day to 7th day of treatment has been increased by 10.2% (*P* < 0.05), indicating that most of the narrow neovascularization has been reduced.

**Conclusion:**

The results and analysis have confirmed that the proposed optimization process by the improved VCA method is both effective and feasible to segment and quantify the NVC with lower noise and fewer artifacts. Thus, it can be potentially applied to monitor the fibrovascular regression during the treatment period.

*Clinical Trial Registration* This trial is registered with the Chinese Clinical Trial Registry (Registered 27 December 2017, http://www.chictr.org.cn, registration number ChiCTR-IPR-17014160).

## Background

Proliferative diabetic retinopathy (PDR), characterized by neovascularization and fibrous hyperplasia, is a common and serious complication of diabetes mellitus (DM) [1]. Persistent vitreous hemorrhage (VH), caused by neovascularization and tractive retinal detachment (TRD), may induce permanent vision loss or blindness that is the most common indication for surgical intervention. Therefore, clear visualization of neovascularization in terms of neovascular complex (NVC) is crucial in diagnosis and treatment monitoring of PDR [2].

Efficient technologies to quantify neovascular changes on NVC in PDR are highly desirable, however, effective choices are fairly limited to date. The traditional color fundus photography has a low resolution that limits its ability for precise quantification [3]. The current “gold standard” to detect diabetic retinopathy is a dilated fundus examination with additional fluorescein angiography (FA), which is utilized to evaluate retinal vasculature in diabetic eye disease [4, 5]. The FA method has high sensitivity in detecting microaneurysms and areas of neovascularization, beneficial to visualize neovascular changes on NVC in diabetic eyes otherwise unobservable for examination; however, it is unable to independently visualize the deep retinal capillary plexus.

In recent years, optical coherence tomography angiography (OCTA), based on depth-resolved motion-contrast imaging, has emerged as a noninvasive imaging modality to image vascular. It can compare fluctuations in signal amplitude caused by relative movement of blood cells to static surrounding tissues [6–8]. In contrast to the traditional dye-based angiography, OCTA image acquisition is much more desirable as information on retinal and choroidal circulation can be almost instantly obtained with higher resolution. In the past decade, substantial research efforts have been dedicated to study the OCTA-based microvascular changes on patients with diabetic retinopathy [8, 9]. Quantitative studies to analyze retinal microvasculature with automated algorithms applied to OCTA have reported reduced vessel density in eyes with diabetic retinopathy [7, 10, 11]. Besides adaptive thresholding on OCTA, other techniques based on Hessian filters have also been proposed in animal studies [12, 13], which have revealed that the estimated vessel radius is very sensitive to pre-selected maximum scale. To lessen the sensitivity in segmentation technique, another segmentation technique, based on intensity value, can be utilized in parallel to the Hessian technique with final segmentation achieved by compounding results. One kind of the artifacts observed in all OCTA approaches is the elongated vessel or tail artifact, i.e., the vessel cross-sections are noncircular line-like [14]. The reason is that the connective eye tissues cause background light and dark changes in imaging, and interferes blood vessel recognition. Thus, rapidly fluctuating speckle signals below the vessels will induce shadow-like artifacts in the angiogram data [15]. Because of these limitations, most currently existing vessel extraction algorithms to segment blood vessels are ineffective for OCTA images. The most recent automatic quantification of vessel densities in OCTA imaging has explored the deep learning method [16], however, it requires a substantial amount of OCTA images that are hard to collect within a short period of time.

In this study, based on an improved vascular connectivity analysis (VCA) algorithm, we propose and demonstrate an optimized approach to segment blood vessels and a combined effort for morphological characterization with noise and artifacts elimination. The length and width of vessels are both obtained in quantitative assessment of the microvascular network. Furthermore, we also present a statistical study for the feasibility of proposed method by monitoring changes of NVC, which is based on OCTA images of PDR patients (N = 14) after treatment by intravitreal injection of conbercept (IVC) [6].

## Methods

### Subjects

This is a single-center prospective, comparative, observational clinical trial conducted in the First Affiliated Hospital of Nanjing Medical University. The trial has been registered with the Chinese Clinical Trial Registry (http://www.chictr.org/cn/ registration number: ChiCTR-IPR-17014160). The study has followed the tenets of the Declaration of Helsinki, and the study protocol has been approved by the Ethics Committee of First Affiliated Hospital of Nanjing Medical University (2015-SR-150). Informed written consents have been obtained from all patients after explaining possible consequences of the study. For this initial study, the OCTA images are provided by authors of Ref. [6] with consensus agreement. While the past study is solely discussing the feasibility of OCTA-based monitoring of neovascular regression on NVC after preoperative IVC, this work is strictly focusing on the image optimization algorithm itself. The demographic and clinical data of the 14 subjects included in the treatment monitoring and statistical analysis section are listed in Table 1.

**Table 1:** The clinical characteristics of the enrolled patients

	Age (years)	Male or female (No.)	Eye (right/left)	Duration of diabetes (years)	IVC treatment
IVC Patients (N = 14)	48 (27–67)	8/6	7/7	8 (2–10)	14

### The OCTA imaging

For the OCTA imaging, AngioVue (version 2017.1.0.151; Optovue, Fremont, CA, USA) has been used to obtain split-spectrum amplitude decorrelation angiography. Two trained examiners (YS and LC) have performed the OCTA examinations after pupil dilatation using the incorporated B-follow-up eye-tracking model. The scanning area is centered on the NVC arising from the optic disc in 6 × 6 mm sections. The navigation line is dragged between retina and NVC, followed by setting the vitreous as reference to exclude retinal vessels underneath. Manual adjustment is occasionally needed to segment retina and NVC on the B-scan model. Low-quality images with signal strength index < 50, images with severe artifacts due to poor fixation, or undetectable images owing to NVC floating too high in the vitreous have been excluded in the analysis. All OCTA examinations have been performed three times for the mean values.

### An improved VCA algorithm for OCTA microvascular extraction and quantification

**Fig. 1:**
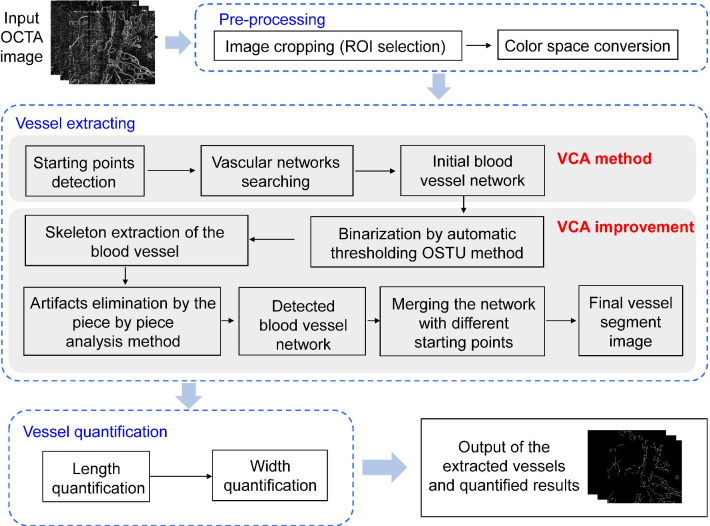
Flowchart of the proposed optimization algorithm method

Based on characteristics of the OCTA blood vessels, an improved blood vessel extraction and quantification method upon the VCA method is proposed with three major parts that include (1) pre-processing, (2) vessel extraction, and (3) vessel quantification. Figure 1 illustrates the three procedures and detailed step-by-step operations. For each processed OCTA microvascular image, a binary image that shows the microvasculars of original image with quantified microvascular parameters is obtained after applying the mentioned processing operations. The first pre-processing part includes image cropping and color space conversion. The next vessel extraction part includes operations of starting points detecting, vascular networks searching, binarization by automatic thresholding OSTU method, skeleton extraction of blood vessels, artifacts elimination, and vascular network merging. The final vessel quantification part includes quantification of the length and width, whereas the length is defined as the cumulative total vessel length and the width is defined as the ratio of cumulative total vessel area over the length. The image processing software used in this study is MATLAB (version R2016a; MathWorks, Inc., Natick, MA, USA). The step-by-step operations are listed in the flowchart of Fig. 1 with details explained next.

In the first pre-processing part, the input image is processed by a two-step process to ensure the subsequent operations simple and effective. The original image is cropped to the region of interest (ROI), and the color space is transformed to gray domain to reduce the computational complexity.

In the second vessel extraction part, the VCA method is first applied to determine the connected area from starting points of blood vessels as the microvascular network. In order to acquire an accurate and optimized vascular network set with lower noise and fewer artifacts, an improved VCA method is hereby proposed. The three main processing steps in the second part are hereby listed.

The first step is to identify the starting points of vascular network. The matrices of OCTA images are traversed by the partial line detection and the number of lines with no effective points are recorded. If proportion of the detected value is lower than the preset threshold, such a point is considered as a starting point. The Z-shaped traversal is then performed and continued to search other starting points of qualified vascular network until all traversal is completed.

The second step is to search all vascular networks connected with starting points. In the beginning, all starting points are marked as part of the initial vascular network and restored in the network point set. Then, we move the detection coordinate from starting points to the next position in the OCTA image, and calculate the minimum distance *d*_*min*_ between the detected point to all points in the set. If *d*_*min*_ is small enough (within 2 pixels), i.e., the detected point is close enough or connected with some vessels restored in the network point set, it will be marked as part of the initial vascular network and stored in the set. Through the global image traversal, all point sets that meet the requirements are stored and the initial blood vessel network is thus obtained. However, since the blood vessels extracted by the regional connectivity method are sensitive to the starting position, the original images will be rotated by 90°, 180°, and 270°, respectively, to search for different starting points and initial blood vessel. Finally, the vascular networks obtained from different starting points are merged to obtain a complete vascular network.

The last step is to optimize the initial microvascular network thus obtained. The binary microvascular image can be achieved by the OTSU image binarization method [17, 18]. In the initial vascular network, some noise and artifacts may be mistakenly marked as vessels due to the distance or gray value too close to the real vessels. Thus, we hereby propose to apply a noise and artifact reduction method, which combines the morphology and piece-by-piece analysis methods, into the VCA. Here, the skeleton of microvasculars in OCTA image is extracted to obtain a thinner vessel graph that presents the vascular skeleton only. The piece-by-piece analysis method is used to evaluate the correctness of extracted vascular skeleton for further noise and artifact reduction. The branch and breakpoint information of each blood vessel curve is used to obtain the branch length and total length of each blood vessel. If the branch length is too short, or the ratio of total length to the number of bifurcation points plus number of breakpoints is lower than the preset threshold, it will be considered as noise or artifacts for elimination. Accordingly, the noise and artifact pixels can easily be distinguished from vessel pixels. A completely optimized vascular network is finally achieved after execution of all mentioned steps.

In the third vessel quantification part with morphological characterization method, the length and width of vessels are quantified. The total area and length of blood vessels are obtained respectively through pixel accumulation from the vessel segment images, and the width is thus defined as the ratio between the area and length.

### Statistical analysis

The data used for statistical analysis are as follows: (1) the quantified parameter length for all involved subjects from the 1st to 7th day after treatment; (2) the quantified parameter width for all involved subjects from the 1st to 7th day after treatment. The statistical term “averaged” throughout the paper represents the arithmetic mean. Unpaired two-tailed independent samples *t*-tests (with Welch’s correction in cases of unequal variances) have been conducted by the GraphPad Prism 7.0 software to evaluate whether the above studies of (1) and (2) may lead to statistically significant difference.

## Results

### The original OCTA image and traditional processing

**Fig. 2:**
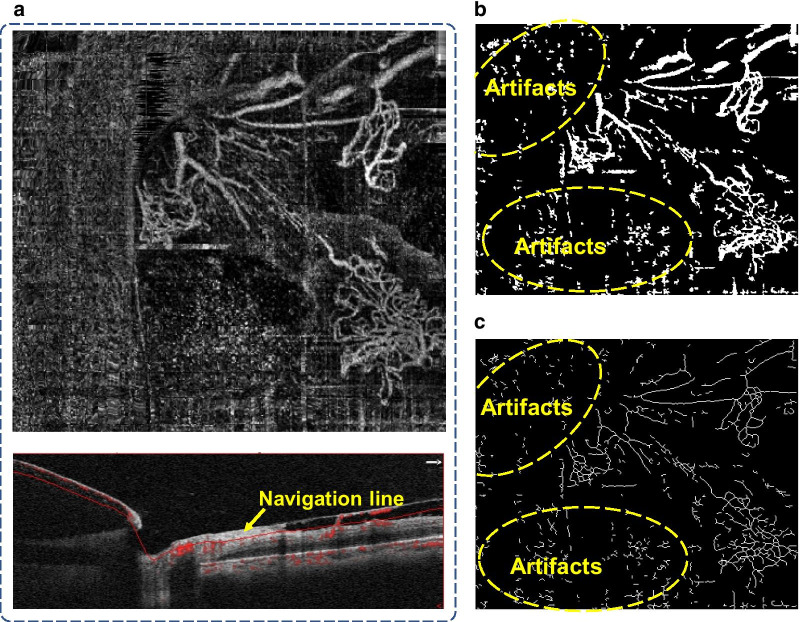
**a** Top left: the original image of neovascularization on NVC floating in the vitreous cavity; bottom left: the corresponding OCT B-scan with flow overlay. **b** The vessel extraction results by the matched filter algorithm. **c** The skeleton of vessels obtained by the skeleton extraction algorithm

The original and processed OCTA images acquired by AngioVue are shown in Fig. 2. The original OCTA image, Fig. 2a, is observed to possess several artifacts and a substantial noise level that results in a fairly low signal to noise ratio (< 50). The vascular network extracted by the traditional matched filtering method [19, 20] is shown in Fig. 2b. Utilizing the blood vessel network and morphological operation, the blood vessel skeleton is extracted and shown in Fig. 2c. Although the matched filtering algorithm is capable of maintaining the blood vessel continuity and obtaining a clear blood vessel skeleton pattern, however, the extracted blood vessels obtained by this traditional method are thicker than the real blood vessels, which would directly alter the accuracy of quantitative blood vessel thickness. In addition, due to the high noise level in the original image, the matched filter method cannot completely eliminate the artifacts and image noise. Thus, the extracted blood vessel network is longer than that of the actual blood vessel, which deteriorates the accuracy in both the blood vessel thickness and length.

### The proposed optimization processes

**Fig. 3:**
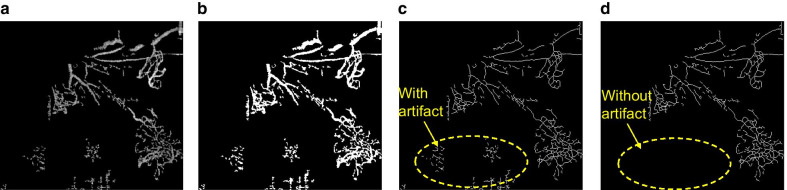
**a** The extraction of vascular network with the improved VCA method. **b** Binarized image of **a**. **c** The skeleton of the vascular network. **d** A noise-free complete skeleton of the blood vessel by the piece-by-piece analysis method

To enhance the image processing accuracy, we again extract and quantify the blood vessels from Fig. 2a utilizing the proposed optimization algorithm, i.e., the improved VCA method, whose details are presented in Sect. 2.3. Originated from Fig. 2a, the re-constructed vascular network that is extracted by the improved VCA method is shown Fig. 3a. To retrieve the length and width information of blood vessels, we also have performed a binarization process upon the Fig. 3a image and the results are shown in Fig. 3b. Furthermore, by utilizing the skeleton extraction algorithm, the skeleton of blood vessel network of Fig. 3b has been acquired and illustrated in Fig. 3c. Compared with the original Fig. 2a image, it is clearly observed that the vascular network is completely extracted in Fig. 3c. However, some nearby noise clusters, as those circled in the yellow mark in Fig. 3c, are still mistakenly identified as blood vessels. Therefore, the last step of the blood vessel analysis method requires a final piece-by-piece analysis for further cleansing these artifacts. The optimized final image after all mentioned processes is presented in Fig. 3d, as we notice, the vascular network is completely extracted and cleaned up with all artifacts and noise removed effectively.

### Treatment monitoring and statistical analysis

**Fig. 4:**
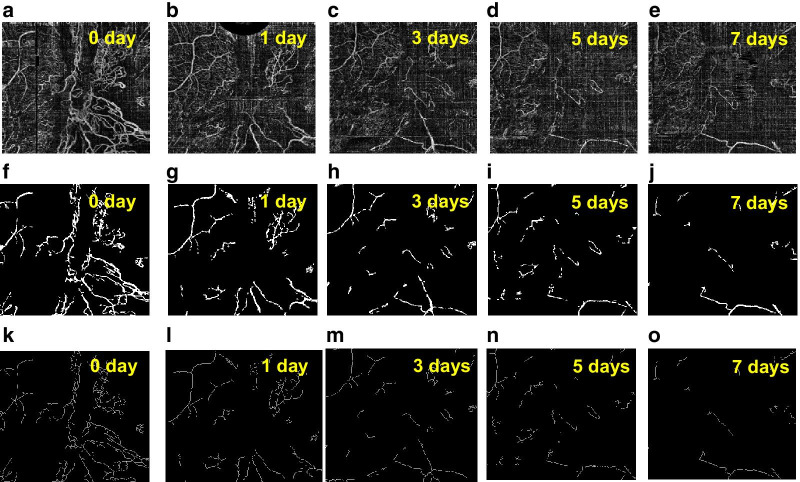
**a** Distribution of the NVC of a sample patient before treatment. **b**–**e** Proliferation of the same region within treatment stages (1, 3, 5, and 7 days respectively). **f**–**j** The binary OCTA images after the improved VCA for neovascularization shown in **a**–**e**. **k**–**o** The corresponding vascular skeleton extraction results for the neovascularization shown in **a**–**e**

**Fig. 5:**
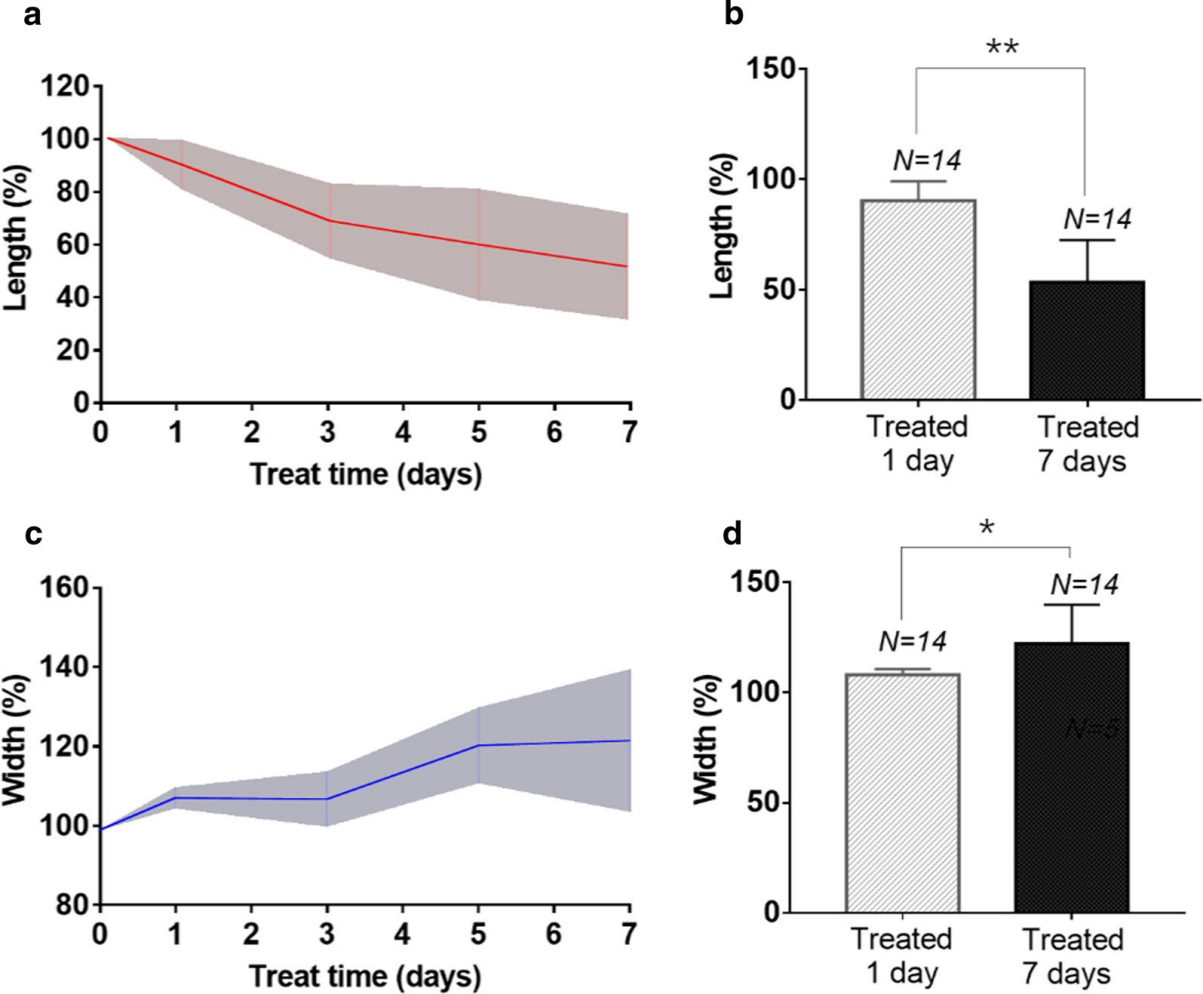
Quantified results of the treatment monitoring of NVC from patients (N = 14) with PDR. **a** The quantified parameter length of vessels as a function of treatment duration for patients (N = 14) with PDR. **b** The mean and standard deviation of the parameter length on the 1st day vs 7th day. **c** The quantified parameter width as a function of treatment duration for patients (N = 14) with PDR. **d** The mean and standard deviation of the parameter width on the 1st day vs 7th day. The *p* value is calculated for each unpaired two-tailed independent samples t-test. ** stands for *p* < 0.01, * stands for *p* < 0.05

In the feasibility study of proposed optimization method in segmentation and quantification of morphological changes of NVC, we have practiced this method to a real treatment monitoring of PDR [6]. To verify the robustness of ongoing conclusion, we have been allowed to access a sample pool of OCTA images of PDR patients (N = 14) to study the statistical significance. The OCTA images of one typical patient with PDR, as an example, are shown in Fig. 4. The results of statistical analysis based on the available sample pool (N = 14) are presented in Fig. 5.

Regarding the example patient, Fig. 4a illustrates the distribution of neovascularization on NVC before treatment applied. Figure 4b–e present the OCTA images of the same region at different treatment stages (1, 3, 5, 7 days, respectively), where the complete proliferating membrane vascular network has been extracted by the optimized VCA algorithm. The corresponding binary OCTA images and vascular skeletons are shown in Fig. 4f–j and Fig. 4k–o, respectively. The processed blood vessel extraction results in Fig. 4 clearly indicate that the distribution range of blood vessels has decreased significantly with days accumulated during treatment.

To statistically evaluate the variation in vascular distribution and thickness, the changes in length and width of blood vessels in vessel network during treatment have been quantified for the patients (N = 14), as shown in Fig. 5. As observed from Fig. 5a, the obtained data show that from the pre-treatment to 1st day of treatment, the averaged length of NVC has been shortened by 9.7%, indicating substantial treatment effectiveness. For the length measurement of all involved subjects, the mean and standard deviation of the 1st day after treatment are compared to those of the 7th day, as shown in Fig. 5b. It shows that the averaged length of blood vessels has continued to decrease by a large reduction of 36.8% (*P* < 0.01) from the 1st to 7th day of treatment. This reduction clearly indicates that the length as a quantified parameter possessing statistically significant difference between the 1st day and 7th day of treatment. Meanwhile, the difference in vessel width between the 1st day and 7th day of treatment, as shown in Fig. 5d, has also been examined. Overall, the averaged width of NVC on the 7th day of treatment has increased to 117.7% ± 12.0% of the pre-treatment value, as illustrated in Fig. 5c. Comparing the averaged width of NVC on the 7th day of treatment to that of the 1st day, it presents an increase of 10.2% (*P* < 0.05). Thus, the statistical results indicate that the width of the neovascularization is enhanced after treatment with statistical certainty.

## Discussion

In comparison with the traditional matched filter results in Fig. 2b, c, the proposed VCA optimization method can efficiently remove a large amount of scattered noise around periphery and obtain a clean vascular network in NVC with the OCTA images. In addition, the improved VCA method can effectively reduce the near-point noise and completely negate any artificial noise, resulting in a much more accurate vascular length and width measurement. Furthermore, in the feasibility study to monitor neovascularization changes in NVC, it indicates that neovascularization has been mostly eliminated after treatment. The supplemental statistical analysis shows that the length of microvascular has been substantially reduced and the width of vessels has increased after treatment. The latter observation, we believe, may due to the fact that some of the smaller vessels have disappeared after treatment and only the larger vessels/larger vasculature persisted, resulting in an increased average width of vascularity as shown in OCTA images. This finding may share a common ground with the regression of exuberant vascular proliferation (EVP) after treatment in OCT images as per previous reports [21, 22]. The quantified results from our image analysis are consistent with the clinical medical observation and diagnosis, which have strongly proved the effectiveness of the proposed VCA improvement. The presented results thus have indicated that the optimized VCA and morphological characterization method have the potential to be applied to quantify and monitor the changes in microvascular on NVC with OCTA images.

One major limitation in this study is that in the final noise and artifacts removal method that is based on a piece-by-piece analysis, it requires a threshold value to identify the noise and artifacts in OCTA images. Thus, it may not be suitable for images with multiple levels of noise and artifacts. Besides, the improved VCA algorithm is required to search the starting points of microvascular in OCTA images from various directions, which leads to computing redundancy and needs further improvement. Thus, studies involving larger-scaled sample patient pools are necessary to further verify the improved VCA algorithm in the near future. Finally, in the busy real-life clinics, it is not very practical to perform a real-time analysis if the algorithm is not directly built into the machine software. To quantize the vascular network in real time with OCTA imaging, the algorithm execution speed shall be further improved and the codes shall be integrated into the software in future.

## Conclusion

In summary, we have proposed and demonstrated an optimized image segmentation and quantification approach to extract vascular network from OCTA images acquired in NVC of patients with PDR. By combining an improved VCA algorithm with the morphological characterization method, we have verified its effectiveness and feasibility with sampled OCTA images and statistical analysis. Comparing with the traditional matched filter method, the optimized VCA method can achieve a final vascular network with lower noise and free of artifacts. Finally, our study of statistical analysis also shows that this approach can be potentially applied in practice to monitor the neovascularization changes after treatment in future with further improvement.

## Data Availability

The datasets during and/or analyzed during the current study available from the corresponding author on reasonable request.

## Abbreviations

NVC: Neovascular complex
OCTA: Optical coherence tomography angiography
PDR: Proliferative diabetic retinopathy
VCA: Vascular connectivity analysis
DM: Diabetes mellitus
VH: Vitreous hemorrhage
TRD: Tractive retinal detachment
FA: Fluorescein angiography
IVC: Intravitreal injection of conbercept
